# Pain After Brachial Plexus Injury Surgery: Variability in Reporting

**DOI:** 10.7759/cureus.102144

**Published:** 2026-01-23

**Authors:** Anna Zakusylo, Aryan Borole, Evan Lobato, Andrew G Baddoo, Diana Vitkovska, Daniel Devine, Jomar N Aryee, Ajul Shah, Brian M Katt

**Affiliations:** 1 Department of Orthopaedic Surgery, Rutgers Robert Wood Johnson Medical School, New Brunswick, USA; 2 Department of Plastic Surgery, Center for Hand and Upper Extremity Surgery, Shrewsbury, USA

**Keywords:** brachial plexopathy, brachial plexus injury, brachial plexus trauma, nerve graft, nerve transfer, traumatic brachial plexus injury, visual analog scale

## Abstract

Pain is a major contributor to morbidity following brachial plexus injury (BPI), yet pain-related outcomes are inconsistently reported. The extent of postoperative pain improvement after surgical reconstruction remains unclear due to variable measurement techniques and nonstandardized reporting intervals. A systematic review was conducted in accordance with the PRISMA guidelines. Studies involving adults undergoing operative treatment for traumatic BPI that reported pain using the Visual Analog Scale (VAS) were included. Data extraction focused on the timing and methods of postoperative pain assessment. Nine studies met the inclusion criteria. Pain reporting demonstrated substantial heterogeneity, with more than 15 different postoperative timepoints reported across studies. Only a small minority of studies reported pain at comparable intervals, precluding pooled analysis. Pain was consistently prevalent after BPI, particularly in cases involving root avulsion, and several surgical techniques were associated with qualitative pain improvement. Overall, pain reporting after BPI surgery lacks standardization, limiting meaningful comparison of outcomes. Standardized measurement intervals and reporting methods are needed to guide clinical expectations and strengthen future research.

## Introduction and background

Traumatic brachial plexus injuries (BPIs) cause devastating motor and sensory deficits in a young, active population. The incidence of BPI has increased with the rise of high-energy trauma. Epidemiological data suggest a growing incidence of BPIs, estimated at 1.6 per 100,000 persons annually in the United States. BPIs are particularly prevalent in urban areas due to the high risk of BPI associated with motorcycle crashes [1]. Although reconstructive strategies have improved motor outcomes, such as elbow flexion and shoulder stability, neuropathic pain remains one of the most disabling consequences of BPI.

Neuropathic pain after BPI arises from peripheral and central mechanisms. Root avulsion injuries, in particular, generate severe deafferentation pain with potential central sensitization. This persistent pain impairs sleep, psychological well-being, functional recovery, and rehabilitation engagement [2]. Yet despite its clinical importance, pain remains underreported in the BPI literature. Several authors have noted that pain outcomes are inconsistently reported in the brachial plexus literature, with most studies emphasizing motor recovery while underrepresenting pain-specific measures [3].

Pain trajectories after surgery are poorly defined. Some studies demonstrate substantial pain improvement with nerve reconstruction or microsurgical dorsal root entry zone (DREZ) lesioning, whereas others report persistent or worsening pain, particularly in complete avulsion injuries [4-8]. The inconsistent measurement of postoperative pain has resulted in a fragmented body of evidence, limiting the ability to counsel patients reliably.

This systematic review was designed to synthesize postoperative pain outcomes following operative treatment of BPI. However, during data extraction, it became clear that the literature is too heterogeneous to support quantitative comparison. Instead, this review describes the variability in pain reporting and highlights the need for standardized reporting guidelines.

## Review

Methods

Search Strategy

A systematic search was performed across PubMed, Embase, and the Cochrane Library following the PRISMA guidelines using keywords related to BPI, surgery, and pain outcomes.

Studies that met the following inclusion criteria were included for review: adult patients (≥18 years), patients who underwent operative treatment for traumatic BPIs, pain outcomes reported using the Visual Analog Scale (VAS), and peer-reviewed articles with extractable data. Studies were excluded from our review if they involved patients treated nonoperatively, included pediatric cohorts, lacked validated pain outcome measurement tools, or lacked extractable postoperative pain data.

Data Extraction and Synthesis

**Figure 1:**
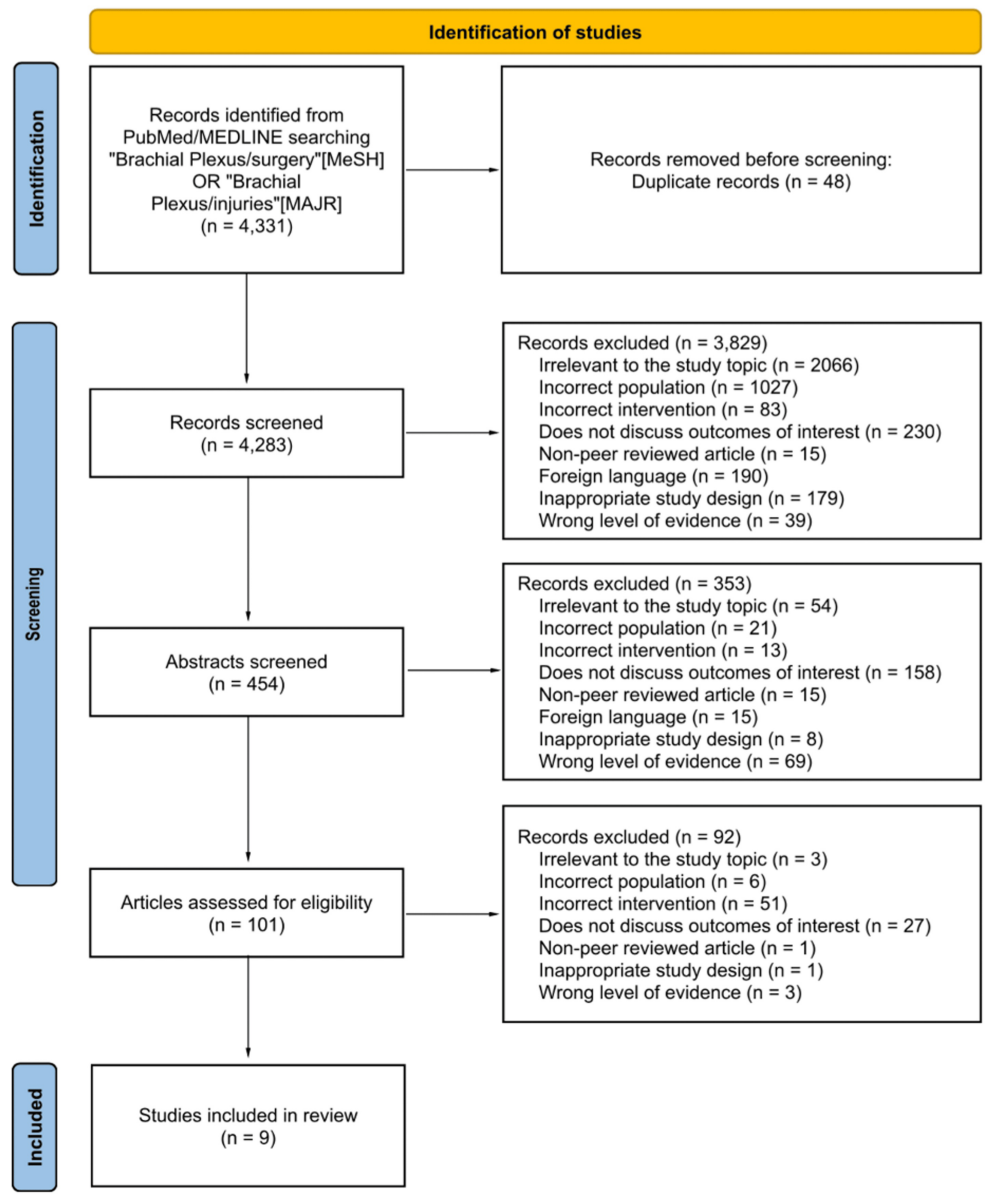
PRISMA flow diagram of the study selection process

Extracted data included injury pattern, surgical technique, pain scale used, and postoperative timepoints. Because of substantial heterogeneity, a pooled meta-analysis was not performed. A descriptive synthesis was produced (Figure 1).

Results

Nine studies met the inclusion criteria (Table 1). These papers represented a wide range of operative treatments for traumatic BPIs, including nerve transfers, nerve grafts, vascularized nerve grafts, free muscle transfers, and dorsal root entry zone (DREZ) procedures. Study sizes varied from small single-surgeon experiences to larger series of more than 100 patients. The follow-up ranged from several months to multiple years. 

**Table 1:** Summary of the included studies

Author (year)	Injury type	Operation	When VAS scores were collected	Pre-op VAS scores	Post-op VAS scores	Sample size
Htut et al. (2006) [5]	Avulsion	Brachial plexus repair with graft and nerve transfer	Mean time of assessment: 4 years after assessment	Not possible to assess most patients prior to surgery, as many were referred early after injury and surgical repairs were performed urgently	2.7	54
	Avulsion	Reimplantation of avulsed nerve roots			4	14
	Avulsion	No repair at all: late referral			4.8	8
Kato et al. (2006) [9]	Avulsion	Brachial plexus repair with graft and nerve transfer (patients grouped based on timing from injury to surgery)	Pre-op scores and time of final VAS assessments not stated	Not stated	Group 1 (<1 m), 2.6; Group 2 (1-3 m), 3.7; Group 3 (3-6 m), 4.0; Group 4 (>6 m), 5.3	148
Armas-salazar et al. (2022) [10]	Injury	Neurolysis (5 roots)	Before surgery and last follow-up (61.9 ± 53.6 months)	8	2.6	3
		Neurolysis (3 roots)		8.25	5	4
		Neurolysis (2 roots)		9	2	3
Gebreyohanes et al. (2023) [11]	Avulsion	Dorsal root entry zone (DREZ)	Median follow-up 37 months	9.1	3.5	10
Maldonado et al. (2023) [12]	Injury	Gracilis muscle transfer	Mean follow-up of 37 months	5.3	4.5	27
Dukan et al. (2023) [2]	Avulsion	Nerve transfer	Pre-op not stated; post-op time at the last follow-up not stated, but inclusion criteria required at least 10 years from surgery	Not stated	3.2	16
Emamhadi et al. (2017) [13]	Avulsion	Nerve transfer	Pain before surgery was compared to that at the six-month follow-up	8.5	0.7	11
Baruah et al. (2024) [14]	Avulsion	DREZotomy	Preop and post-op VAS scores	7.3	3.8	18
Dong et al. (2012) [15]	Avulsion	DREZ lesioning	Pre-op and 12 months post-op	8.9	0.9	7

Pain reporting was inconsistent across the literature. Investigators measured pain at more than 15 different postoperative intervals. Some included very early postoperative scores, others reported short- or mid-range follow-up at three or six months, and many provided only a single final follow-up value. The timing of this final measurement ranged widely, from less than six months to more than five years after surgery. Only a small number of studies reported pain at comparable timepoints, and even those differed in scale or reporting format.

The structure of pain reporting also varied. Some authors provided mean values with standard deviation. Others reported medians or ranges. Several studies included pain only in figures or narrative descriptions without extractable numerical data. A number of papers listed preoperative and postoperative values but failed to specify when the postoperative measurement was collected. Because of these inconsistencies, the pain data could not be combined or analyzed quantitatively.

Even with the heterogeneity, several broad descriptive trends were visible. Pain after BPI was common across nearly all cohorts. Many patients continued to report burning, aching, or neuropathic symptoms after surgery. Studies that stratified by injury pattern consistently found that more severe injuries, particularly root avulsions, exhibited higher preoperative pain and more persistent symptoms after reconstruction. Many reports also described some degree of postoperative improvement in pain, but the variation in measurement methods and follow-up intervals prevented comparison of the magnitude or timing of that improvement.

Overall, pain outcomes across these studies show consistent evidence of postoperative symptoms but substantial inconsistency in how results are measured and reported. These descriptive findings set the stage for interpreting the underlying trends observed in the wider literature.

Discussion

The primary finding of this review is that pain reporting after brachial plexus surgery is profoundly inconsistent. Although pain is a major determinant of disability and overall quality of life, the literature does not use a uniform approach to measuring or reporting pain outcomes. In our review, we found an additional 35 studies that utilized standardized instruments, such as Disabilities of the Arm, Shoulder and Hand (DASH) and Numerical Rating Scale (NRS); however, the sample sizes were significantly smaller compared to those using VAS, with widely variable collection timepoints, making comparison unfeasible. Because of this substantial heterogeneity in both measurement tools and follow-up timepoints, these studies were excluded from our analysis entirely. The heterogeneity making study comparison difficult also makes it challenging to counsel patients with confidence about expected postoperative pain trajectories.

Multiple studies highlight the heavy pain burden faced by patients with root avulsion injuries. Bertelli et al. reported that 64% of individuals with avulsion injuries experienced substantial preoperative pain, and many continued to report persistent symptoms after surgery [16]. 

Surgical technique also appears to influence postoperative pain. Authors have reported improvements with neurotization. DREZ procedures have shown consistent benefit for refractory neuropathic pain. Chen et al. reported a decrease in VAS scores from >9 preoperatively to just >3 at final follow-up among patients with long-standing avulsion pain [17]. Vascularized ulnar nerve grafts produced improvements in pain and disability scores in the cohort reported by Lin et al. [18]. Combined contralateral C7 transfer with free functioning muscle transfer also produced symptomatic improvement, with moderate or mild relief noted in the majority of treated patients [19]. Although the degree of improvement cannot be compared across studies because of inconsistent timing and inconsistent scoring methods, the overall direction of improvement was similar.

Pain trajectories varied widely across the included studies. Some authors noted early improvement shortly after surgery, while others described more gradual reduction in symptoms that appeared only when patients began to demonstrate reinnervation or functional return. Several investigations commented that patients who recovered useful motion tended to experience greater pain improvement than those who did not. Although this relationship cannot be quantified based on available data, it suggests that motor recovery and pain reduction may follow similar paths in a subset of patients.

Despite broad agreement that pain is important, this review highlights the remarkably inconsistent ways in which pain outcomes are recorded in the literature. Investigators used different scales, different intervals, and different reporting formats. Some provided clear preoperative and postoperative values, others reported single timepoints without context, and many used graphical formats without extractable numbers. This limits the ability to identify typical pain patterns or compare the effectiveness of different surgical techniques. Future work will need to adopt more uniform pain reporting methods to better understand recovery trajectories and guide clinical expectations.

## Conclusions

Pain is a major and persistent consequence of BPI, but postoperative pain outcomes are reported with substantial inconsistency across the literature. Because few studies use standardized scales at comparable timepoints, meaningful pooled analysis is not possible. Standardized pain reporting is urgently needed to improve the ability to compare outcomes, guide treatment decisions, and provide more reliable expectations for patients undergoing reconstruction.
